# Syntheses of APTMS-Coated ZnO: An Investigation towards Penconazole Detection

**DOI:** 10.3390/ma15228050

**Published:** 2022-11-15

**Authors:** Elvira Maria Bauer, Gabriele Bogliardi, Cosimo Ricci, Daniele Cecchetti, Tilde De Caro, Simona Sennato, Alessandro Nucara, Marilena Carbone

**Affiliations:** 1 Institute of Structure of Matter, Italian National Research Council (ISM-CNR), Via Salaria km 29.3, 00015 Monterotondo, RM, Italy; 2 Department of Chemical Science and Technologies, University of Rome Tor Vergata, Via della Ricerca Scientifica, 00133 Rome, RM, Italy; 3 Institute of Nanostructure Materials, National Research Council (ISMN-CNR), Via Salaria km 29.3, 00015 Monterotondo, RM, Italy; 4 Institute of Complex Systems, Italian National Research Council (ISC-CNR) Sapienza Unit, and Physics Department, Sapienza University, P.le A. Moro 5, 00185 Rome, RM, Italy; 5 Department of Physics, Sapienza University, P.le A. Moro 5, 00185 Rome, RM, Italy

**Keywords:** coated ZnO, APTMS, extrinsic chemiluminescence, penconazole detection

## Abstract

Extrinsic chemiluminescence can be an efficient tool for determining pesticides and fungicides, which do not possess any intrinsic fluorescent signal. On this basis, (3-aminopropyl) trimethoxysilane (APTMS)-coated ZnO (APTMS@ZnO) was synthesized and tested as an extrinsic probe for the fungicide penconazole. Several synthetic routes were probed using either a one-pot or two-steps method, in order to ensure both a green synthetic pathway and a good signal variation for the penconazole concentration. The synthesized samples were characterized using X-ray diffraction (XRD), infrared (IR), Raman and ultraviolet-visible (UV-Vis) spectroscopy, scanning electron microscopy (SEM) imaging and associated energy-dispersive X-ray (EDX) analysis. The average size of the synthesized ZnO nanoparticles (NPs) is 54 ± 10 nm, in line with previous preparations. Of all the samples, those synthesized in two steps, at temperatures ranging from room temperature (RT) to a maximum of 40 °C, using water solvent (G-APTMG@ZnO), appeared to be composed of nanoparticles, homogeneously coated with APTMS. Chemiluminescence tests of G-APTMG@ZnO, in the penconazole concentration range 0.7–1.7 ppm resulted in a quenching of the native signal between 6% and 19% with a good linear response, thus indicating a green pathway for detecting the contaminant. The estimated detection limit (LOD) is 0.1 ± 0.01 ppm.

## 1. Introduction

Chlorinated pesticides and fungicides, such as tebuconazole, tetraconazole, and penconazole are routinely used for pest control in cultivated vegetables, herbs, and spices [1,2]. Their maximum residual level in the final commercialized product is subjected to restrictions, which depend on the country where they are marketed [3], with consequent implementation of monitoring programs [4]. In addition, in the case of imports to the European Union, the control is performed in designated countries, on a random basis, regardless of the access or destination points. Consequently, there is a time delay between the borders being crossed and the test outcome, during which the transport of the food proceeds normally. In cases where pesticide residual is too high, the contaminated food is destined to be destroyed, regardless of how far it has travelled. The tests for organochlorinated pesticide (OCPs) levels are usually performed by HPLC [5,6,7,8,9] and several implementations have been proposed to improve on selectivity and detection limits [10,11,12].

HPLC requires pre-extraction which is lengthy and expensive [13], in spite of the effort that has been dedicated to the optimization of the procedure [14,15]. The drawbacks of HPLC analysis of pesticides have stimulated the implementation of alternative techniques, suitable for fast and possibly on-field detection, especially since a quicker assessment of OCPs levels would minimize the waste occurring due to late reports of maximum residual level (MRL) violations and would aid more efficient procedures of quality and safety checks. In this framework, surface-enhanced Raman spectroscopy (SERS) has been proposed as a possible alternative for OCPs detection. This technique is based on the enhancement of the Raman signal of a molecule induced by metallic nanostructures, particularly gold and silver colloidal nanoparticles [16,17,18], which have proved to be very versatile [19,20] and sometimes are used in combination with metal oxides such as ZnO [21]. When employed for the detection of OCPs, however, linkers are necessary between the substrate and target, due to the highly hydrophobic structures of the latter and their low affinity toward the SERS substrates. Therefore, diamines [22,23], dithiols [24], bipyridinium dications [25], carbon, and metal-organic-frameworks [26] have been used to improve the selectivity. However, issues remain concerning the stability and reliability [16].

Additional detection methods with potential in-field developments are based on the employment of fluorescence. Since OCPs are “silent”, i.e., do not possess inherent fluorescence, their detection relies on the induction of extrinsic fluorescence, which can be modulated as a function of the pesticide concentration.

The choice of the material to be employed as the extrinsic fluorescent probe is crucial for an efficient pesticide detection and for the environmental impact of its productions, its future disposal, and its bioaccumulation [27]. In this regard, materials such as CdTe dots, although fluorescent, are best discarded for pollution reasons, since the leaching of cadmium and tellurium from the dots may represent an environmental hazard [28]. This is especially the case if the dots are employed as free detecting agents and not encapsulated, for instance in solar panels. Carbon materials for sensing [29,30,31,32], especially quantum dots, are also an option, although issues of selectivity may arise, due to the lack of specific surface anchoring. In order to promote environmentally friendly alternatives, biocompatible materials such as ZnO [33] are proposed for the chemiluminescent core [34,35,36,37,38,39] for detection purposes. ZnO is generally recognized as a safe (GRAS) substance by the US Food and Drug Administration (USFDA) [40]. Moreover, ZnO exhibits excellent UV absorption and photoluminescent properties due to its wide bandgap, which makes ZnO a suitable candidate as a chemiluminescent probe [41,42,43].

Subsequent coating with a silanizing agent may cause an interaction with selected pesticides and chemiluminescence variation. The pathway for determining a *green profile* or a *greenprint* for pesticide detection includes selecting efficient synthetic preparation strategies for detecting particles with minimal environmental impact.

Synthesis methods of metal oxides and mixed oxides (materials used in several types of detection systems [44,45,46,47,48,49,50,51,52,53,54,55]) are varied and may be conveniently used to tune the structural, morphological, and optical properties [56,57,58,59,60].

In the present paper, we propose a trial system, tested in aqueous solvent, which employs coated ZnO nanoparticles for the detection of penconazole. In particular, ZnO chemiluminescence is used as the probe for monitoring concentration variation.

As for the coating, silanes were selected, in particular aminopropyltrimethoxy silane, because of their potential to bind to the ZnO on one side, and interact with chlorinated pesticides on the other side, through the amine functional group. Furthermore, when used for *p*-nitrophenol detection in aqueous solutions, the analogue aminopropyltriethoxy silane (APTES)-coated ZnO displayed a larger fluorescence signal variation than bare ZnO, thus indicating an enhanced coating-related effect [61].

APTES is often used as a coating agent as well as a template, due to its cross-linking properties which ensure the creation of a uniform shell around the core [62,63,64]. Furthermore, the presence of the -NH_2_ terminal is a hook for interactions with the -Cl of the organochloride pesticides. In the present paper, we focus on the synthetic strategies for achieving coated ZnO in conditions as environmentally friendly as possible. Therefore, we used the APTMS analogue as our coating agent, since it has methoxy instead of ethoxy ligands around Si, i.e., slightly better leaving groups and likely allows milder synthetic conditions. We probed one-pot and two-steps syntheses and found that the best synthetic strategy for APTMS-coated ZnO (APMTS@ZnO) is a two-steps synthesis, carried out at 40 °C or lower temperatures, depending on the stage of the reaction, fully in water solvent and with good coverage of the ZnO surface.

Tests of chemiluminescence as a function of the penconazole concentration indicate a good linear response in the detection range 0.7–1.7 ppm, with a LOD of 0.1 ppm. The advantages of the system we are proposing are twofold: on the one hand, we aim for a simple and green strategy for a synthesis of coated ZnO nanoparticles, on the other hand, the synthesized material will be used for the detection of pesticides such as penconazole by using a routine technique, i.e., a fluorescence which can also be applied in-field [65,66].

## 2. Materials, Equipment, and Synthetic Procedures

### 2.1. Materials and Equipment

Zinc acetate dihydrate (Zn(ac)_2_∙2H_2_O), zinc nitrate hexahydrate (Zn(NO_3_)_2_∙6H_2_O), potassium hydroxide (KOH), sodium hydroxide (NaOH), anhydrous absolute ethanol (EtOH), nitric acid (HNO_3_, 65%), (3-aminopropyl)trimethoxysilane (APTMS), and penconazole were purchased from Sigma-Aldrich (St. Louis, MO, USA). Toluene was purchased from Scharlab (Barcelona, Spain). The water used in all the experiments was doubly distilled and purified using a Milli-Q system (Millipore, Burlington, MA, USA).

Infrared spectra were recorded with a Shimadzu Prestige-21 FT-IR instrument (Shimadzu, Kyoto (Japan)), equipped with an attenuated total reflectance (ATR) diamond crystal (Specac Golden Gate), in the 400–4000 cm^−1^ range, with a resolution of 4 cm^−1^.

The samples were characterized by XRD diffraction, using an X’ pert pro X-ray diffractometer by Philips (Amsterdam, The Netherlands), operated with CuK-Alpha radiation.

The Raman spectra were acquired on a Renishaw 2000 equipped with a Peltier cooled CCD camera (Renishaw, New Mills (UK)) and a Leica optical microscope, operated at 785 nm. Prior to sample evaluation, automated alignment and calibration for each laser and grating pair were undertaken to ensure proper instrument performance.

UV-visible spectra were carried out with a PerkinElmer Lambda 950 spectrophotometer (Perkin Elmer, Inc., Waltham (MA, USA)), using quartz cuvettes with a 1 cm optical path.

The surface morphology of the synthesized particles was determined with an FE-SEM, Field Emission Scanning Electron Microscope SUPRA TM 35, Carl Zeiss SMT (Oberkochen, Germany), operating at 7 kV, with the Energy Dispersive Microanalysis (EDS/EDX, INCAx-sight, Model: 7426, Oxford Instruments, Abingdon, Oxfordshire, UK), operating at 20 kV.

A Malvern Nano-ZetaSizer, equipped with a 5 mW HeNe laser (λ = 632.8 nm) and thermostated cell, was used to perform both dynamic light scattering (DLS) and electrophoretic mobility measurements. For DLS, this instrument employs a backscattering configuration, i.e., the scattered light is collected at an angle of 173°. This detection geometry offers the advantage of being less sensitive to multiple scattering effects than the more conventional collection angle of 90°. DLS autocorrelation functions were analyzed by using the cumulant method [67]. The first cumulant was used to calculate the diffusion coefficient D of the particles, which is converted into the hydrodynamic diameter, D (D = 2R, radius) by applying the Stokes–Einstein relationship D = kBT/3πηD, where kBT is the thermal energy and η is the solvent viscosity. The polydispersity index was determined by the second cumulant.

To determine the electrophoretic mobility, the Doppler shift was analyzed using the phase analysis light scattering (PALS) method [68]. The mobility µ of the particle is converted into a ζ-potential using the Smoluchowski relation ζ = µ η/ε, where ε and η are the permittivity and the viscosity of the solution, respectively.

For both DLS and electrophoretic measurements, the result represent the average and the standard deviation on three different measurements, where each measurement is composed of 12 sub-runs, at least.

Chemiluminescence spectra were obtained on a Perkin Elmer (Waltham, MA, USA) LS50B spectrophotometer, equipped with a Xenon discharge lamp and working in the dual-monochromator configuration, step 0.1 nm. Samples were housed in quartz cuvettes with a 1 cm optical path. Excitation and emission slits were selected at 5 nm. For the chemiluminescence quenching tests, 1 mg/10 mL water dispersions of G-APTMS@ZnO were sonicated for 30 min, prior to the measurements. Furthermore, different quotas of penconazole were dissolved in 0.5 mL ethanol and added to the water dispersion of G-APTMS@ZnO, in order to achieve the target concentrations. The blank measurements were carried out on 1 mg/10 mL water dispersions of G-APTMS, additioned with 0.5 mL ethanol, to achieve the same water–ethanol ratio as in the measurements containing penconazole.

### 2.2. Synthetic Procedures

Two synthetic procedures were followed, one-pot and two-steps. For the two-steps procedure, 12.5 mmol of zinc nitrate hexahydrate were dissolved in 125 mL ultrapure water and kept under vigorous stirring at 40 °C by means of an oil bath. 125 mL of a 0.1 M NaOH solution were added dropwise, and the slurry was aged at 40 °C under stirring overnight. The white precipitate was separated from the mother liquor by centrifugation at 3500 rpm for 10 min, washed twice with distilled water, resuspended in water, transferred into a Petri glass dish, and left drying in an oven at ~40 °C overnight.

The subsequent coatings with APTMS were carried out according to two different procedures. In the first one, 100 mg of ZnO were dispersed in 10 mL ultrapure water by sonication for 30 min in a 100 mL round-bottom flask. Afterwards, 40 μL APTMS were added, and the dispersion was sonicated for further 30 min. The dispersion was refluxed under stirring overnight (T = 100 °C). The sample was centrifuged twice, at 3500 rpm for 10 min, rinsing first with water and then with ethanol, and left drying overnight at ~40 °C, to achieve the N-APTMS@ZnO.

The second procedure was fully carried out at RT. More specifically, 100 mg of ZnO were dispersed in 10 mL ultrapure water sonicating for 30 min in a 100 mL round-bottom flask. Drops of a 2 M solution of HNO_3_ were added until reaching a pH of 6.50. After stirring for further 30 min, 40 μL of APTMS were added. The pH rose to 8.64 and then stabilized at 8.22. The dispersion was left under stirring at RT for 24 h. The sample was centrifuged twice, at 3500 rpm for 10 min, rinsing first with water and then with ethanol, and left drying overnight, in an oven at ~40 °C, to achieve the G-APTMS@ZnO.

The one-pot synthesis with Zn(ac)_2_ was carried out by dissolving 1.88 mmoles Zn(ac)_2_ 2·H_2_O in 10 mL of anhydrous absolute ethanol, in a round-bottom flask immersed in an oil bath, pre-heated at 68 °C and kept under vigorous stirring for 90 min. 5 mL of a 0.8 M solution of KOH was added dropwise and the white turbid reaction mixture was left under stirring for 1 h at RT. Finally, 60 μL of APTMS were diluted in 5 mL ethanol and added dropwise, followed by the addition of 0.5 mL of ultrapure water. The reaction mixture was left under stirring for 120 min. The slurry was rinsed twice with toluene in a separating flask, centrifuged twice at 3500 rpm for 10 min, rinsed with ethanol, and finally dried overnight at RT, to achieve the S-APTMS@ZnO.

## 3. Results and Discussion

To achieve a green synthetic pathway of APTMS@ZnO nanoparticles with chemiluminescent properties, different strategies were probed.

Two main approaches were followed, one-pot and two-steps, using two different sources of Zn, i.e., Zn(ac)_2_ or Zn(NO_3_)_2_. The former is employed in sol-gel types of synthesis [69], the latter in precipitations.

In the one-pot approach, Zn(ac)_2_ and APTMS are added into the same reaction vessel, to synthesize ZnO nanoparticles and simultaneously silanize them (sample S-APTMS@ZnO).

In the two-steps approach, the ZnO nanoparticles are synthesized according to the reaction:

$${\mathrm{Zn}(\mathrm{NO}}_{3}{)}_{2}+2\mathrm{NaOH}\;\overset{T_{P}}{\to }{\mathrm{Zn}(\mathrm{OH})}_{2(s)\;}{,\;\mathrm{ZnO}}_{\left(s\right)}{+2\mathrm{NaNO}}_{3(\mathrm{aq})}\overset{T_{D}}{\to }{\;\mathrm{ZnO}}_{\left(s\right)}{+H}_{2}{O+2\mathrm{NaNO}}_{3(\mathrm{aq})}$$

T_P_ and T_D_ were both set at 40 °C, which proved to be the lowest temperature at which ZnO could be achieved without further calcination [70]. ZnO nanoparticles are subsequently coated with APTMS in a different reaction pot, at any convenient moment.

Two different silanization procedures were followed, at different temperatures and pHs (samples N-APTMS@ZnO and G-APTMS@ZnO).

The summary of the employed conditions for the synthesis of the coated ZnO nanoparticles is reported in Table 1.

The samples characterization was carried out by IR, Raman, UV-Vis spectroscopies, XRD, SEM, EDX, DLS, and zeta-potential measurements in order to determine their crystallographic phase, the efficacy of the silanization, the presence of residuals, the morphology, the size, and the absorbance and fluorescence properties. Finally, chemiluminescence quenching by penconazole was performed on the most “promising” sample as a function of the pesticide concentration.

**Figure 1:**
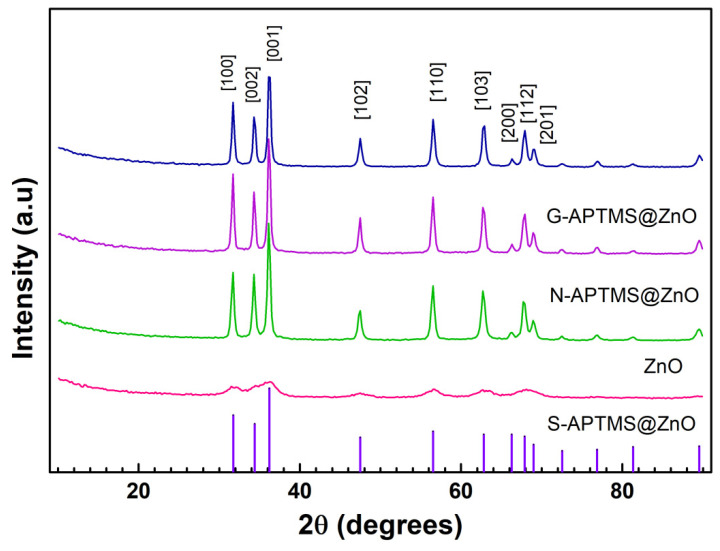
XRD pattern of ZnO and the coated samples N-APTMS@ZnO, G-APTMS@ZnO, and S-APTMS@ZnO. The violet vertical lines represent reflexes from the JCPDS 73-1520 reference card of hexagonal ZnO. The planes indexes are also reported.

Powder X-Ray diffraction patterns for all the samples are reported in Figure 1. For comparison purposes, the reflexes of pure ZnO are additionally reported from JCPDS card 73-1520, corresponding to a hexagonal close-packed wurtzite structure. There is a one-to-one correspondence between synthesized ZnO and the reference reflexes, thus indicating the achievement of the target compound and the reproducibility of the synthetic procedures fully carried out at 40 °C [70]. To go into further detail, reflexes at 2θ = 31.7°, 34.3°, 36.1°, 47.5°, 56.5°, 62.9°, 66.3°, 67.9°, and 68.9° were identified as belonging to the (100), (002), (001), (102), (110), (103), (200), (112), and (201) planes.

The subsequent silanization of ZnO performed in the two-steps procedures does not affect the cores of the particles, whose XRD patterns are comparable to the uncoated sample [71]. Nonetheless, the spectra are compatible with the presence of surface layers of silanes, which would not contribute to the diffraction patterns.

The one-pot synthesis of S-APTMS@ZnO provides features specific to ZnO, whose comparatively broader and lower intensity appearance can be ascribed to multiple factors, including small particle size, size polydispersion, random mutual orientation of the particles, a degree of amorphism, and a thick layer of coating [72].

**Figure 2:**
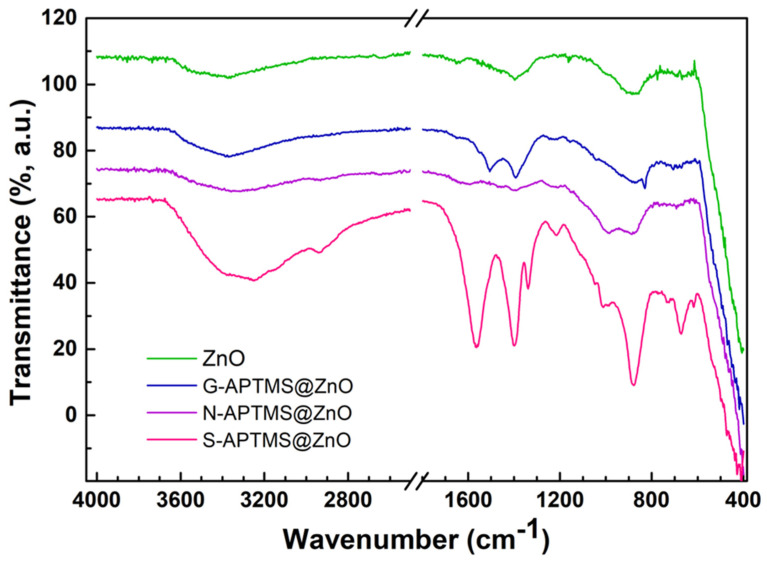
IR spectra of ZnO and of the coated samples N-APTMS@ZnO, G-APTMS@ZnO, and S-APTMS@ZnO, in the range 4000–400 cm^−1^.

The efficacy of the silanization procedure was evaluated, in the first instance, by IR spectroscopy. The spectra corresponding to the various samples are reported in Figure 2. The main peaks along with their assigments are reported in Table 2. The IR spectrum of ZnO has the typical steep band rising from 400 cm^−1^ towards higher frequency values, with a shoulder at 543 cm^−1^ assigned to Zn-O stretching vibrations [73,74]. Additional broad bands around 860 cm^−1^ and 3360 cm^−1^ are assigned to -OH libration and stretching vibration, respectively, and are associated with the presence of Zn(OH)_2_ on the surface of the ZnO nanoparticles [73,74]. Adsorbed water molecules also contribute to the high frequency band. Additional bands at 1394 cm^−1^ and 1651 cm^−1^ are attributed to the presence of surface carbonate groups and/or carboxilic -C=O groups [75]. This is likely the outcome of a carbonation process owing to the CO_2_ in the air, since the synthesis is carried out in an open vessel. Upon silanization, new features appear, related both to the presence of APTMS and to the condensation bonds between APTMS and ZnO. The relative intensity of the IR features reflects the condensation mechanism as well as the extent of the coating layers. More precisely, the G-APTMS@ZnO sample is characterized by a broad band centered at 3300 cm^−1^, to which stretching vibrations of -OH, -NH_2_ -CH_2_ and -CH_3_ contribute. At lower frequencies, the region between 1100 cm^−1^ and 1600 cm^−1^ presents the -NH_2_ bending as well as -CH_2_ scissoring of the aminopropyl and methoxy moieties [76], although the latter overlaps with the carbonate split-stretching band at the same frequency. The region 1050–800 cm^−1^ carries the signature of the silanization process, i.e., Si-O-Si and above all the Zn-O-Si vibrations [77]. Finally, below 800 cm^−1^, a few additional characteristic bands appear, -NH_2_ wagging and Si-C stretching.

The N-APTMS@ZnO sample has a similar spectrum as G-APTMS@ZnO, the main difference being the presence of a more intense peak at 993 cm^−1^, related to Si-O-Si stretching vibrations and the absence of the Si-O-CH_3_ band, thus indicating a larger silane network. S-APTMS@ZnO shows much more intense and well-resolved features and it presents distinguishable bands related to -OH, -NH_2_, and -CH_3_/-CH_2_, stretching vibrations at high frequencies. The strong peaks at about 1564 cm^−1^, 1400 cm^−1^, and 1330 cm^−1^ are attributed to C=O stretching and C-O stretching, respectively [69,78], and are related to the presence of acetate groups complexed with a metal like zinc. This is partly due to the confinement of KAc into the ZnO structure during the synthesis [79]. Additional silanization features below 1000 cm^−1^ are also present. The overall intensity of the bands also suggests a thicker coating. In general, all samples present a certain silanization degree. However, some differences can be observed among the various samples, which can ultimately be ascribed to slightly different ZnO-APTMS condensation mechanisms. In particular, the Si-O-Si and the Zn-O-Si bands have nearly the same intensity in the N-APTMS@ZnO sample, whereas the Si-O-Si band is dimmer in the G-APTMS@ZnO sample, suggesting a less extensive network of Si-O-Si bonds. Simultaneously, the narrow band of Si-O-CH_3_ bending only appears for the G-APTMS@ZnO sample.

**Figure 3:**
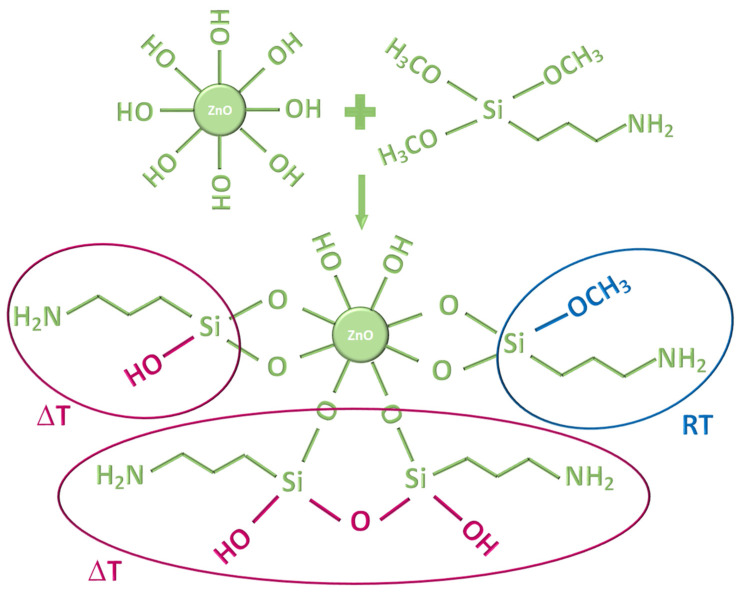
Sketches of ZnO condensation with APTMS. The red oval shapes outline the hydroxylation procedure and the Si-O-Si network formation at higher temperatures (reflux). In the blue circle, the persistence of -OCH_3_ moiety upon RT synthesis is outlined.

As for the mechanism, in their study on ZnO silanization via ZnO-APTES interaction, Nicolay et al. [77] proposed that the condensation occurs between the surface Zn(OH)_2_ layer covering the ZnO particle and the alkoxy moiety of the APTES molecule. However, we hypothesize that the condensation pathways may be different depending on the reaction temperature. At a higher temperature (i.e., the temperature of the reactions carried out under reflux), hydrolysis of the methoxy moiety may occur with consequent hydroxylation of the silane. Water elimination from adjacent hydroxyls leads to a further Si-O-Si network. When silanization reactions are carried out at RT, as is the case for G-APTMS@ZnO, the silane hydroxylation occurs to a lesser extent, with a consequently lower degree of Si-O-Si network and a larger amount of methoxy moieties on the coated sample. This is in line with the observed sharp intensity at 829 cm^−1^ relating to the methoxy moiety and with the relatively low intensity of the Si-O-Si band at 1000 cm^−1^, for the G-APTMS@ZnO sample. The products of the condensation reactions are sketched in Figure 3.

**Figure 4:**
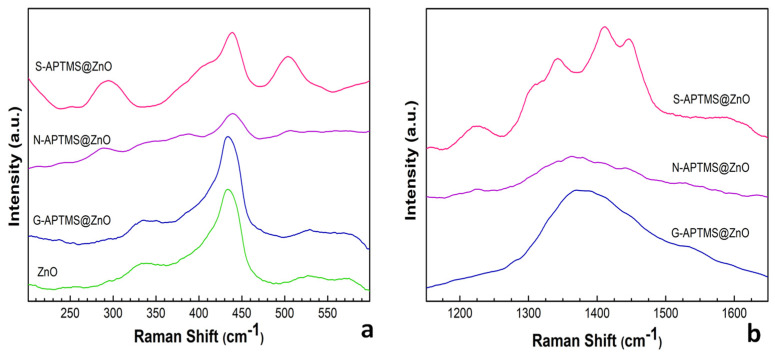
Raman spectra of ZnO and the coated samples N-APTMS@ZnO, G-APTMS@ZnO, and S-APTMS@ZnO. Panel (**a**) 200–600 cm^−1^, panel (**b**) 1100–1700 cm^−1^.

Raman spectra of the samples were acquired using a laser excitation at 785 cm^−1^ and are reported in Figure 4, in the ranges 200–600 cm^−1^ (panel a) and 1100–1700 cm^−1^ (panel b). These ranges are characteristic of ZnO-related features and of terminal -NH_2_ groups, respectively. More precisely, the spectrum of the synthesized ZnO displays features at 332 cm^−1^, 434 cm^−1^, and 578 cm^−1^ which can be attributed to the optical modes, E_2_ (Transversal Optical—TO), E_2_ (Longitudinal Optical—LO) and E1, respectively [80]. A low peak is present at 534 cm^−1^ which is not assigned [80]. The spectrum of the G-APTMS@ZnO in this energy range is similar to that of pure ZnO. The spectra of N-APTMS@ZnO and S-APTMS@ZnO present additional features at 290 cm^−1^ and 505 cm^−1^, more pronounced for the latter, as well as a shift of the main peak, which appears at 438 cm^−1^. Both features are related to the presence of acetate, and especially the lower energy one can be attributed to ν (ZnO), due to the presence of Zn(ac)_2_ [81,82] either as reagent in excess (S-APTMS@ZnO) or as newly formed compound (N-APTMS@ZnO). The higher-energy feature is compatible with the optical absorption of acetate [83] as well as with the formation of overlayers. In the region 1100–1700 cm^−1^, both G-APTMS@ZnO and N-APTMS@ZnO are characterized by a broad feature centered at 1380 cm^−1^, which is considered diagnostic of terminal -NH_2_ attached to an alkyl moiety for the analogous APTES-coated ZnO nanoparticles [80] and attributed to the -NH_2_ rocking vibration. S-APTMS@ZnO is characterized by features at 1225 cm^−1^, 1307 cm^−1^, 1341 cm^−1^,1412 cm^−1^, and 1447 cm^−1^. The features at 1341 cm^−1^ and 1447 cm^−1^ have been assigned to -CO symmetric stretching and -CH_3_ symmetric bending of the acetate [84], whereas the other ones can be ascribed to APTMS overlayers [80].

**Figure 5:**
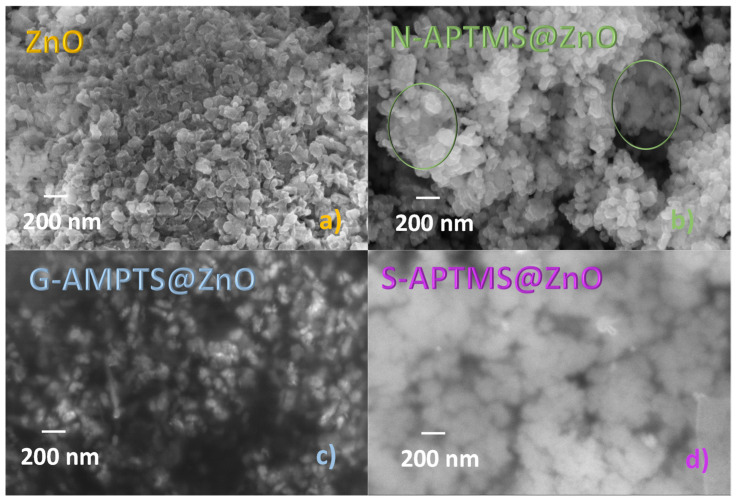
SEM images of coated and uncoated ZnO at 100 K magnification: (**a**) ZnO, (**b**) N-APTMS@ZnO, (**c**) G-APTMS@ZnO, (**d**) S-APTMS@ZnO.

The morphology and average size of the samples was investigated using SEM microscopy and representative images are reported in Figure 5. ZnO synthesized and dried at 40 °C displays the typical 54 ± 10 nm average size, in line with previous observations for this type of preparation procedure [70]. Upon silanization, the nanoparticle morphology is largely preserved. Depending on the sample, various degrees of sheathing are observed, which can be ascribed to the presence of the APTMS coating on the ZnO. More specifically, the N-APTMS@ZnO presents only limited regions of sheathed surface, marked with green circles in Figure 5b. The ZnO nanoparticles appear embedded in the sheath in the G-APTMS@ZnO sample (Figure 5c), although the particle shapes can still be distinguished. The nanoparticles also appear immersed in the sheath in the S-APTMS@ZnO sample and the particle contours can no longer be recognized, as if the silane formed a multilayer on top of the particles.

An estimate of the composition of the coated samples has been calculated using EDX analysis and the results are reported in Table 3. The atomic ratios between Zn/O, Zn/C, Zn/N, and N/Si are fairly similar for G-APTMS@ZnO and N-APTMS@ZnO, whereas they are significantly lower for S-APTMS@ZnO, thus indicating a greater coverage of the ZnO nanoparticles by the silane.

Overall, the one-pot synthesis seems to produce ZnO samples coated with a greater amount of APTMS, but also more agglomerate.

The zeta-potential and DLS measurements were carried out on all samples and are reported in Table 4. The zeta-potential of ZnO is 25.8 ± 0.70 mV, i.e., within a range where high electrostatic repulsive forces exist between the nanoparticles, with consequently good stability in aqueous solutions [85]. This value increases upon silanization, according to the order N-APTMS@ZnO < G-APTMS@ZnO < S-APTMS@ZnO, which also reflects the degree of coating observed in the SEM images. The associated DLS measurements indicate an average diameter in the 175–225 nm range. These values are apparently larger than the average nanoparticle size estimated by SEM. However, in the comparison it must be taken into account that DLS assesses hydrodynamic ranges, which are typically larger, and that agglomerates may form in water dispersions. More precisely, this concerns the interplay of a few parameters, such as the size, concentration, shape, polydispersity, and surface properties of the nanoparticles [86]. The average dynamic radius decreases with the increasing surface charge, likely due to the surface repulsion induced by the charge, with the exception of the S-APTMS@ZnO. This sample, however, has large inhomogeneity, as also demonstrated by the large polydispersity index.

**Figure 6:**
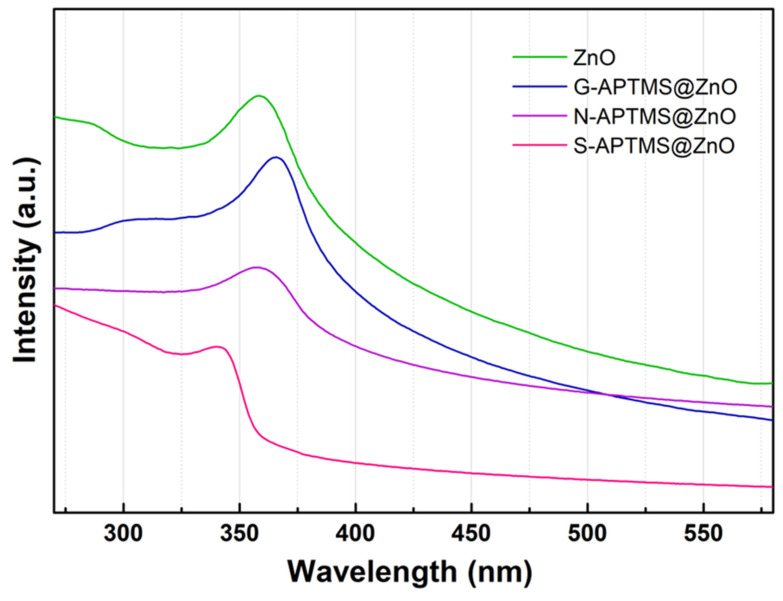
UV-visible spectra of G-APTMS@ZnO, N-APTMS@ZnO, and S-APTMS@ZnO.

The UV-visible spectra of the synthesized samples in water dispersions are displayed in Figure 6. They are all characterized by a main excitonic peak in the 300–400 nm range. The absorption peak of ZnO is centered at 358 nm and is compatible with hexagonal wurtzite [87]. G-APTMS@ZnO has a main peak at 366 nm, N-APTMS@ZnO at 357 nm, and S-APTMS@ZnO at 340 nm.

The corresponding band gaps can be estimated using the equation Eg (eV) = (hc/λ_m_) = 1240/λ_m_ (nm), where Eg is the optical band gap, h is the Planck’s constant, c is the speed of light and λ_m_ is the wavelength of maximum absorption [88,89]. Band gaps and λ_m_ are recorded in Table 5, along with literature data about ZnO synthesized using different methods, and whose UV-visible spectra were collected from dispersions in solvents. A main exitonic peak at 378 nm is observed for the water dispersion of ZnO nanoparticles with spherical shape in the range 30–90 nm. They were obtained by reacting Zn(NO_3_)_2_·6H_2_O and egg white, followed by calcination at 600 °C [88]. An exitonic peak at 368 nm was observed in the case of ethanol dispersion of a mix of nearly spherical and rod-shaped ZnO aggregates, obtained by Zn(ac)_2_ thermolysis. In our case, the excitonic peak of ZnO is observed at 358 nm, i.e., it is blue-shifted. In general, this is attributed to confinement effects, which occur when the average size of the nanoparticles decreases, and it is compatible with the smaller size of our samples as compared to the ones in the literature. Taking the ZnO value of 358 nm as a reference, the effect of the coating is threefold, depending on the synthesis procedure. In particular, the excitonic peak is nearly unaffected in the case of N-APTMS@ZnO, likely due to the scattered coverage of the ZnO surface by the silane as observed by SEM imaging. The excitonic peak (and band gap) is blue-shifted in the case of S-APTMS@ZnO and it is red-shifted in the case of G-APTMS@ZnO.

**Figure 7:**
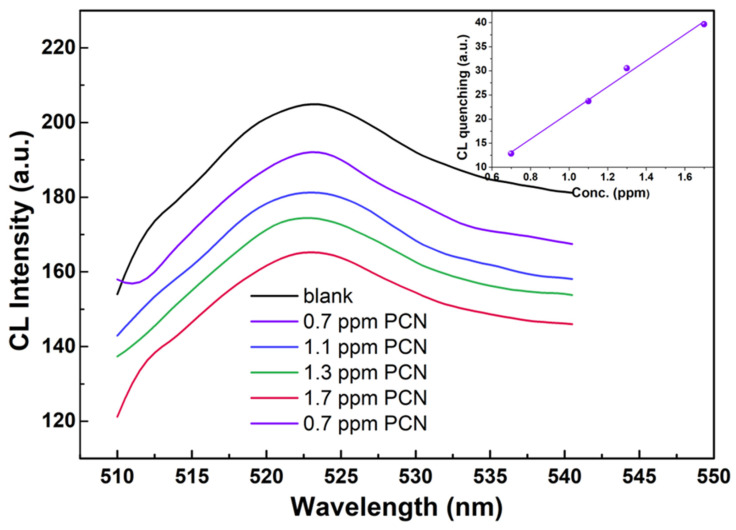
Chemiluminescence spectra of G-APTMS@ZnO as a function of penconazole (PNC) concentration. The peak intensity variation is reported in the inset as a function of the concentration, indicating a good linear response.

The blue shift of the capped S-APTMS@ZnO can be related to further confinement effects, due to the size reduction and the formation of anisotropic morphology initiated by the thick silane layer which gives rise to edge-dependent optical properties [90,91]. The red shift is typically associated with the insertion of defects such as the migration of the Zn interstitial position and it is likely induced in the ZnO lattice by room-temperature silanization. Overall, of the synthesized coated samples, N-APTMS@ZnO is not homogeneously coated (SEM images) and S-APTMS@ZnO has traces of the initial acetate reagent (IR and Raman characterizations). G-APTMS@ZnO, on the other hand, not only presents a homogeneous coating, but it is also the sample synthesized in the blandest conditions, at temperatures ranging from room temperature to 40 °C max, and by employing water solvent only. Therefore, further tests on penconazole detection were carried out on G-APTMS@ZnO. The outcome is reported in Figure 7. By using an excitation wavelength of 365 nm, the chemiluminescence of G-APTMS@ZnO is observed at 523 nm. The quenching effects as a function of the penconazole concentration were probed in the range 0.7–1.7 ppm. The corresponding peak intensity variation range lies between 6% and 19%, with a good linear response, as indicated in the inset of Figure 6.

The LOD was evaluated as the concentration corresponding to three times the standard deviation of the signal in the blank sample and it was found to be 0.1 ± 0.01 ppm. This value is in line with LODs found for the detection of OCPs by SERS (detection of penconazole using this technique has not been reported so far, and we made a comparison with other OCPs), and higher than for HPLC detection of penconazole (Table 6).

## 4. Conclusions

In the present paper, one-pot and two-steps synthetic strategies were pursued in order to achieve APTMS-coated ZnO nanoparticles with chemiluminescent properties, for the detection of the fungicide penconazole. The low-temperature synthesis, fully carried out in water, i.e., in green reaction conditions, appeared to be the most effective for obtaining APTMS@ZnO, defined by a ZnO core, nanoparticles dimensions averaging 54 ± 10 nm, a homogenous layer of coating, and no trace of residual reagents. This sample is also characterized by a red shift of the band gap and by the presence, to some extent, of the terminal methoxy moiety. The tests of chemiluminescence as a function of the penconazole concentration indicate a linear response in the probed range 0.7–1.7 ppm and a LOD of 0.1 ± 0.01 ppm.

## Figures and Tables

**Table 1:** Summary of the synthesis conditions. Ovn = overnight.

Sample	Step	Salt	[Zn^2+^]	Base	[OH^−^]	APTMS	ZnO	Solv.	Temp.	Time	pH
ZnO	1st	Nitrate	0.1 M	NaOH	0.1 M			H_2_O	40 °C	ovn	
G-APTMS@ZnO	2nd					40 μL	0.125 M	H_2_O	40 °C	24 h	6.5/8.22
N-APTMS@ZnO	2nd					40 μL	0.125 M	H_2_O	100 °C	ovn	
S-APTMS@ZnO	Single	Acetate	0.19 M	KOH	0.8 M	60 μL/5 mL EtOH		EtOH	68 °C	2 h	

**Table 2:** Assignment of the IR features. Sh = sharp, st = strong, m = medium, v st = very strong, w = weak, vw = very weak, sd = shoulder, br = broad, ν = stretching, δ = bending, ω = wagging, χ = scissoring.

ZnO-cm^−1^	S-APTMS@ZnO-cm^−1^	N-APTMS@ZnO-cm^−1^	G-APTMS@ZnO-cm^−1^	Assignment
3360 br	3387 sd			-OH ν
		3330 br	3330 br	-OH ν + -NH ν + -CH_2_ ν
	3248 br			-NH ν
	2935 m-w	2934 w		-CH_2_ ν
		1598 w		-NH_2_ δ
	1564 v st			-C=O (C-O) ν
			1550 sd	-NH_2_ δ
1510			1506 st	-CO_3_^2−^ ν_as split_
		1454 vw		-CH δ
1394 br				-CO_3_^2−^ ν_as_
	1400 v st			C-O (C=O) ν
		1398 w	1392 st	-CH_2_ χ
	1339 st			-C-O (C=O) ν
	1215 m	1217 w	1215 w	-C-O- ν NH_2_ ω
	1015 w	993 m	1039 sd	Si-O-Si ν
	878 st	881 m	881 m	Zn-O-Si ν
860 br				Zn-OH libr
			829 sh	Si-O-CH_3_ ν
	672 st	668 vw	670 vw	NH_2_ wag SiC str
	617 w			-CH δ
543 sd	543 sd	543 sd	543 sd	Zn-O ν

**Table 3:** EDX analyses of the coated ZnO samples. Atomic percentages of C, N, O, Zn, and Si averaged over three different areas for each sample. In addition, the atomic ratios between the various elements are reported.

Sample	C	N	O	Zn	Si	K	Zn/O	Zn/C	Zn/N	N/Si
G-APTMS@ZnO	15.0	1.4	45.0	38.0	0.6	/	0.8	2.5	27.1	2.3
N-APTMS@ZnO	20.1	1.6	40.1	37.1	1.1	/	0.9	1.8	23.2	1.5
S-APTMS@ZnO	53.2	2.1	29.2	13.2	2.0	0.3	0.5	0.2	6.3	1.05

**Table 4:** DLS and zeta-potential measurements. 2R = Average hydrodynamic diameter; PDI = Polydispersity index.

Sample	2R (nm)	PDI	Z Pot (mV)
ZnO	214.4 ± 6.9	0.182 ± 0.032	25.8 ± 0.70
N-APTMS@ZnO	201.0 ± 2.5	0.161 ± 0.018	26.8 ± 0.30
G-APTMS@ZnO	175.7 ± 3.5	0.148 ± 0.011	27.4 ± 0.75
S-APTMS@ZnO	226.0 ± 47.0	0.449 ± 0.054	38.4 ± 0.50

**Table 5:** Exitonic peaks and estimated band gaps of the synthesized samples and literature references.

Sample	λ_m_-nm	Band Gap-eV	Ref.
ZnO Water	378	3.28	[88]
ZnO EtOH	368	3.37	[89]
ZnO	358	3.46	this work
G-APTMS@ZnO	366	3.39	this work
N-APTMS@ZnO	357	3.48	this work
S-APTMS@ZnO	340	3.65	this work

**Table 6:** LODs of penconazole detected by HPLC in various matrices and OCPs by SERS. LE = Liquid Extraction, DAD = Diode Array Detector, ES = Enantioselective.

Sample	Pesticide	Matrix	Method	LOD (ppm)	Ref.
G-APTMS@ZnO	Penconazole	Water	Fluorescence	0.1	This work
Au/Ag	Dieldrin	Water	SERS	0.3	[24]
Ag NPs sheets	Lindane	Water	SERS	0.0872	[24]
Ag	Endosulfan	Water	SERS	0.167	[24]
Ag/Au	HCH	Water	SERS	1	[92]
	Penconazole	Water	HPLC	2.5 × 10^−3^	[14]
	Penconazole	Grape, Tea	ES-HPLC	0.3–1.5 × 10^−3^	[10]
	Penconazole	Peach, Plum	HPLC/DAD	0.1 × 10^−3^	[11]
	Penconazole	tobacco	LE-GC	0.011	[12]
